# Following Nerve Injury Neuregulin-1 Drives Microglial Proliferation and Neuropathic Pain via the MEK/ERK Pathway

**DOI:** 10.1002/glia.21124

**Published:** 2011-01-06

**Authors:** Margarita Calvo, Ning Zhu, John Grist, Zhenzhong Ma, Jeffrey A Loeb, David L H Bennett

**Affiliations:** 1 Wolfson CARD, Kings College LondonHodgkin Building, Guys Campus, SE1 1UL, London, United Kingdom; 2 Department of Neurology, Center for Molecular Medicine and Genetics, Wayne State University School of MedicineDetroit, Michigan

**Keywords:** microgliosis, neuregulin-1, erbB receptors, ERK1/2, AKT, neuropathic pain

## Abstract

Following peripheral nerve injury microglia accumulate within the spinal cord and adopt a proinflammatory phenotype a process which contributes to the development of neuropathic pain. We have recently shown that neuregulin-1, a growth factor released following nerve injury, activates erbB 2, 3, and 4 receptors on microglia and stimulates proliferation, survival and chemotaxis of these cells. Here we studied the intracellular signaling pathways downstream of neuregulin-1-erbB activation in microglial cells. We found that neuregulin-1 *in vitro* induced phosphorylation of ERK1/2 and Akt without activating p38MAPK. Using specific kinase inhibitors we found that the mitogenic effect of neuregulin-1 on microglia was dependant on MEK/ERK1/2 pathway, the chemotactic effect was dependant on PI3K/Akt signaling and survival was dependant on both pathways. Intrathecal treatment with neuregulin-1 was associated with microgliosis and development of mechanical and cold pain related hypersensitivity which was dependant on ERK1/2 phosphorylation in microglia. Spinal nerve ligation results in a robust microgliosis and sustained ERK1/2 phosphorylation within these cells. This pathway is downstream of neuregulin-1/erbB signaling since its blockade resulted in a significant reduction in microglial ERK1/2 phosphorylation. Inhibition of the MEK/ERK1/2 pathway resulted in decreased spinal microgliosis and in reduced mechanical and cold hypersensitivity after peripheral nerve damage. We conclude that neuregulin-1 released after nerve injury activates microglial erbB receptors which consequently stimulates the MEK/ERK1/2 pathway that drives microglial proliferation and contributes to the development of neuropathic pain. © 2011 Wiley-Liss, Inc.

## INTRODUCTION

Microglial cells are the resident immune cells of the central nervous system. They display remarkable plasticity and can change their physiology in response to environmental cues. Following injury they proliferate and migrate to accumulate in regions of neuronal degeneration and produce a wide variety of pro-inflammatory molecules (reviewed in Inoue and Tsuda, 2009; Scholz and Woolf, 2007). Production of such pro-inflammatory substances including IL-1β, TNF-α, NO, BDNF, or IL-6, can further recruit microglia, activate astrocytes, and increase neuronal excitability (reviewed in Milligan and Watkins, 2009). Injury to a peripheral nerve results in a marked microgliosis within the dorsal horn of the spinal cord and this contributes to the development of neuropathic pain (Tsuda et al., 2003). In such situations where the blood brain barrier is not disrupted (Abram et al., 2006; Lu et al., 2009) proliferation and migration of resident microglia is likely to be the principal means by which microglial numbers increase (Ajami et al., 2007). Therefore signals within microglia that enhance microglial proliferation and chemotaxis appear as potential targets to modulate the excessive inflammatory response and potentially the development of neuropathic pain. Neuregulin 1 (NRG1)-erbB signaling has recently been identified as one such target (Calvo et al., 2010).

NRG1 is one of a family of growth factors (*NRG1-4*) which has a key role in neural and cardiac development (Gassmann et al., 1995; Lee et al., 1995; Meyer and Birchmeier, 1995), it can modulate synaptic plasticity (reviewed in Mei and Xiong, 2008) and stimulate the proliferation, survival and motility of a number of different cell types. We have recently shown that NRG1 is a survival, proliferative and chemotactic factor for microglia *in vitro* and in addition can promote the release of Il-1β from these cells. Treatment *in vivo* with intrathecal NRG1 induces cold and mechanical pain related hypersensitivity (Calvo et al., 2010; Lacroix-Fralish et al., 2008). Peripheral nerve injury results in the activation of NRG1-erbB signaling specifically within microglia contributing to the development of microgliosis and consequently neuropathic pain (Calvo et al., 2010).

Through alternative splicing, the *NRG1* gene produces numerous isoforms which include both secreted and transmembrane forms (which can undergo further proteolytic processing to be released from the cell membrane, (reviewed in Esper et al., 2006; Newbern and Birchmeier, 2010). All isoforms have an EGF-like domain that is critical for mediating biologic activity and which binds to the tyrosine kinase receptors erbB3 and 4. These receptors, subsequently heterodimerize with erbB2 which lacks a ligand binding domain but which is a key co-receptor in mediating signal transduction (Carraway and Cantley, 1994). Within an activated receptor dimer, the C-terminal regulatory tail is *trans*-autophosphorylated on tyrosines and recruits downstream signaling molecules that contain phosphotyrosine-binding Src homology-2 (SH2) domains. Intracellular signaling pathways which have been demonstrated to be subsequently activated include the extracelullar signal regulated kinases (ERK), the p38 mitogen-activated protein kinases (p38 MAPK), and the phosphatidylinositol-3-kinase (PI3K)/Akt pathway. These can modulate distinct aspects of the cellular response for instance ERK signaling promotes cell proliferation and PI3K/Akt cellular motility (Eckert et al., 2009; Maurel and Salzer, 2000; Sei et al., 2007) in response to NRG1.

Microglia express all three erbB 2, 3, and 4 receptors (Calvo et al., 2010; Dimayuga etal., 2003; Gerecke et al., 2001). The intracellular signaling cascades downstream of NRG1-erbB signaling within these cells are however unknown. Both the ERK and p38 MAPK pathways have previously been shown to be activated within microglia of the dorsal horn following peripheral nerve injury (reviewed in Ji et al., 2009) making them interesting candidates for mediating NRG1 effects. Here we demonstrate that NRG1 which is released after nerve injury and signals via the erbB receptors activates the mitogen-activated ERK-regulating kinase (MEK)/ERK pathway in microglia. This leads to an increase in proliferation of these cells and a proinflammatory phenotype that induces the development of neuropathic pain.

## MATERIALS AND METHODS

### Animals and Surgery

Adult male Wistar rats were used in accordance with UK Home Office regulations. Nerve injury was produced by tight ligation and transection of the left L5 spinal nerve. Briefly, animals were anaesthetized using a mixture of medetomidine hydrochloride (0.25 mg/kg) and ketamine (60 mg/kg) administered in a single intra-peritoneal injection. The animals were placed in the prone position and under sterile conditions a paramedial incision was made to access the left L4-L6 spinal nerves. Approximately one-third of the L6 transverse process was removed. The L5 spinal nerve was identified and carefully dissected free from the adjacent L4 spinal nerve and then tightly ligated using 6-0 silk and then transected distally to the suture. The muscle layer was sutured, and the wound was closed with Vicryl 3-0. To label dividing cells, rats were injected with 5-bromo-2**′**-deoxyuridine (BrdU; Sigma dissolved in 0.007N NaOH/PBS, 100 mg/kg body weight i.p.) 24 hours before perfusion and fixation.

### Intrathecal Injections

Injections were performed by lumbar puncture using the method described by Mestre et al. (1994). Under isofluorane anaesthesia a 26G needle from an insulin syringe (Myjector U-100 Terumo) was inserted between the L5 and L6 vertebrae, where the cord consists mainly of spinal roots. A volume of 10 or 20 μL was injected at a constant speed after which the needle was slowly removed. The quality of each injection was ensured by the observation of an injection-induced tail-flick.

### Drugs and Delivery

Neuregulin β1 EGF domain (rHRGβ1 aa176-246, R&D Systems cat no. 396HB) was intrathecally administered at 0.4 or 4 ng dissolved in sterile saline in a volume of 20 μL. Injections were repeated every 24 hours and the animals were sacrificed at day 4. In another experiment NRG1 was administered to naive animals together with the MEK inhibitor U0126 (Promega) or the inactive analogue U0124 (Merck) which were dissolved in 24% DMSO and saline (as previously described by Zhuang et al., 2005). For this experiment a syringe was filled with 10 μL of inhibitor or the inactive analogue (10 μg) and 10 μL of NRG1 (4 ng) which were separated by a small air bubble. The injections were performed once daily four times. Behavioral tests were done 24 hours after each injection for the first 3 days, and the animals were sacrificed at day 4. In other experiments we administered U0126 or U0124 (10 μg in 20 μL) daily through lumbar punctures to animals which underwent a L5 spinal nerve ligation.

For continuous intrathecal delivery of the erbB2 inhibitor PD168393 we inserted an intrathecal catheter that was connected to an Alzet osmotic pump (Cupertino, model 2002) filled with the inhibitor. Briefly, a laminectomy of the L2 vertebra was performed, the dura was cut and a soft catheter was inserted into the subarachnoid space of the spinal cord. PD 168393 (Calbiochem) an irreversible erbB inhibitor was dissolved in 5% DMSO and delivered intrathecally at 10 μg/day. Control animals were given the same vehicle solution lacking the active compound. To sequester endogenous NRG1, we used a fusion protein (HBD-S-H4, Ma etal., 2009) that was injected intrathecally once at the time of surgery (3 μg in 20 μL of sterile saline per injection).

The drug doses were selected on the basis of previous reports and our preliminary studies. Before surgery animals were randomly allocated into experimental study groups (computer-generated randomization schedules). Operators and data analysts were blinded throughout the study.

### Behavioral Testing

Mechanical withdrawal thresholds were tested using a Dynamic Plantar Aesthesiometer (Ugo Basile, Italy) which is an automated version of the von Frey hair assessment. A maximum cut-off of 50 g was used. The withdrawal threshold is calculated as the average of three consecutive tests with at least 10 min between each test. To measure cold allodynia, we applied a drop of acetone to the plantar hindpaw and measured the time that the animal spent licking, shaking, or lifting the paw during the following 2 min (Kontinen and Dickenson, 2000). All behavioral tests were performed by an investigator blinded to randomization schedule.

### Histology

After defined survival times, animals were terminally anaesthetized and transcardially perfused with 4% paraformaldehyde plus 1.5% picric acid in 0.1M phosphate buffer. The lumbar spinal cords were excised, cryoprotected in 20% sucrose, cryostat cut (20 μm) and thaw-mounted onto glass slides. Spinal cord sections were incubated overnight with the primary antibody: rabbit anti-phospho-p38MAPK (1:100, Cell Signalling), rabbit anti-phosphoAkt (1:100, Cell Signalling) or rabbit anti-phospho-ERK (1:500, Cell Signalling), all of which were viewed by tyramide amplification (TSA™ Biotin System, Perkin Elmer) For co-localization studies the slides were then incubated with rabbit anti-Iba1 (1:1,000, WAKO). Following primary antibody incubation sections were washed and incubated for 1.5 hours with corresponding secondary antibody solution (Extra-Avidin FITC 1:500 or Cy3 1:400, both from Stratech, UK). Slides were washed, cover-slipped with Vectashield mounting medium (Vector Laboratories) and visualised under a Zeiss Axioplan 2 fluorescent microscope (Zeiss, UK). Antibody detection of BrdU incorporated into DNA requires pre-treatment of the tissue to expose the BrdU epitope. For this purpose we used the antigen retrieval method described previously (Tang et al., 2007). Primary antibody solution contained mouse anti BrdU (1:200, BD:Biosciences) plus rabbit anti Iba-1 (1:1,000, Wako) and the Secondary Antibody solution contained corresponding IgG-conjugated FITC 1:200 plus IgG-conjugated Cy3 1:400 (both from Stratech, UK). Microglia cells were 4% PFA fixed for immunohistochemistry. Microglia were identify using Iba1 antibody (1:1,000, WAKO). Proliferation in microglial cells was assessed using BrdU 10 μM which was administered 15 hours before fixation. Antigen retrieval was achieved by denaturing DNA with 2N HCl incubation for 30 min at 37°C, and neutralizing the acid by immersing sections in 0.1M borate buffer (pH 8.5) for 10 min. Cells were then ready for staining using the same protocol previously described.

### Primary Microglia Cell Culture

Mixed glial cultures were isolated from cortex of P3 Wistar rats according to the method of Giulian and Baker (Giulian and Baker, 1986). After mechanical and chemical dissociation cells were seeded in DMEM with 10% FBS at a density of 500,000 cells/mL and cultured at 37°C in humidified 5% CO_2_/95% air. All reagents used were purchased from Invitrogen. Medium was replaced every 2 to 3 days and confluency was achieved after 5 days *in vitro*. Confluent mixed glial cultures were manually shaken for 5 min and the floating cells were pelleted and subcultured. This method resulted in 96% to 99% purity as assessed by Iba1 and DAPI staining. Cells were incubated overnight in standard medium to allow them to attach firmly to the coverslips. The next day they were incubated in serum free medium for 2 to 4 hours to ensure these cells were in a resting or basal condition before using them for experiments. Neuregulin β1 EGF domain (rHRGβ1 R&D Systems) was used (10 nM) for survival, proliferation and chemotaxis assays. Each assay consisted in at least three independent experiments each of which had every condition applied in triplicates. For survival assays microglial cells were suspended in serum-free DMEM and treated with the PI3K inhibitor Wortmannin (1 μM, Sigma) or the MEK inhibitor U0126 (10 μM, Promega) or 0.1%DMSO (vehicle) alone or with NRG1 10 nM and left for 3 days before fixation and staining. For proliferation assays microglial cells were suspended in 5% FBS DMEM and treated in the same way.

### Chemotaxis Assay

Chemotaxis was assessed using the Boyden chamber (Neuroprobe, Bethesda, MD). Polycarbonate filters (5 μm pore) were installed in the chamber, whose bottom wells were filled with serum-free DMEM with or without NRG1 (10 nM). Freshly prepared microglia were suspended in serum-free DMEM and were pre-treated for 1 hour with the different kinase inhibitors (Wortmannin 1 μM or U0126 10 μM or 0.1%DMSO). Then they were placed into the top wells (50,000 cells/well) of the Boyden chamber and left in a CO2 incubator at 37°C for 3 hours. The filter was removed; the cells on the top side of the filter were wiped off and the filter with the remaining migrated cells was fixed with Methanol for 10 min and stained with RapiDiffII (Biostain RRL, UK). Photomicrographs were acquired under light microscopy (Axioskop X-cite 120, Zeiss, Germany) and the number of cells that had migrated to the bottom side was counted.

### Western Blots

Animals were sacrificed using terminal anaesthesia and transcardially perfused with 0.9% saline to wash out all blood. The L5 dorsal horns were rapidly removed and dissected using “open book” method (the spinal segment was cut into a left and right half from the ventral midline and each half was further split into the dorsal and ventral horn at the level of the central canal) and then quickly frozen in liquid nitrogen. Microglial cultures or spinal cord dorsal horn were homogenized in NP40 lysis buffer (20 mM Tris, pH 8, 137 mM NaCl, 10% glycerol, 1% NP-40, 2 mM EDTA), 20 μM leupeptin, 5 mM sodium fluoride, 1 mM sodium orthovanadate, 1 mM PMSF, and protease inhibitor cocktail). The lysate were spun at 13,000 rpm at 4°C for 15 min and the protein concentration of supernatant was determined using a BCA Protein Assay kit (Thermo Scientific). Proteins (50 μg/tissue lysate, 25 μg/cell lysate) were separated using 8% or 10% SDS-PAGE, and transferred to nitrocellulose membranes. Membranes were then blocked in 10% skimmed milk in PBS-T (PBS + 0.1% Tween 20) for 1 hour at room temperature. Membranes were incubated with primary antibody, rabbit phospho-Akt (1:1,000), rabbit Akt (1:1,000), rabbit phospho-Erk (1:2,000), rabbit Erk (1:2,000), rabbit phospho-p38 (1:500), rabbit p38 (1:500), diluted in 5% BSA in TBS-T (TBS + 0.1% Tween 20, all from Cell Signalling) with gentle shaking at 4°C overnight. After washing with PBS-T for five times and 5 min each time, membranes were incubated with donkey anti-rabbit HRP-conjugated secondary antibody (1:10,000; GE Healthcare) for 1 hour at room temperature. After several PBS-T washes as described above membranes were revealed using ECL-plus reagent (GE Healthcare) for 5 min for detection by autoradiography.

### Quantification and Analysis

For immunohistochemistry analysis quantitative assessment was carried out by determining the numbers of immunoreactive cells within four areas of 10,000 μm^2^ in the superficial dorsal horn on five to seven randomly selected L5 spinal sections from each animal. For BrdU staining the whole dorsal horn was analyzed. Microglia in which process length was less than double the soma diameter were classified as presenting an effector morphology. Microglia in which the process length was double the soma diameter were classified as surveying (previously called resting) cells (Stence et al., 2001). Cells were sampled only if the nucleus was visible within the plane of section and if cells profiles exhibited distinctly delineated borders. All analyses were performed with the operator blinded to treatment groups. For Western Blots analysis, films were scanned with Cannon Scanner N1240U. Intensity of specific pERK and ERK bands (ERK1 44 kDa, and ERK2 42 kDa), as well as phospho-Akt and Akt (60 kDa) and phospho-p38 and p38MAPK (38 kDa) were quantified using Adobe Photoshop 7.0.1. The ratio between phosphorylated protein and total protein was obtained. This ratio was normalized against control and expressed as fold increase.

### Statistical Analysis

Sample sizes for experiments were based on results from pilot studies. Data sets were tested for normality using the Kolmogorov-Smirnov test and for homogeneity of variance using Levene's Test. Parametric or non parametric tests were used accordingly. Behavioural data was analyzed using RM two-way ANOVA. *P* < 0.05 was considered as significant. Data are presented as mean ± SEM.

## RESULTS

### NRG1 Treatment Induced Phosphorylation of ERK1/2 and Akt Without Activating p38MAPK

**Fig. 1:**
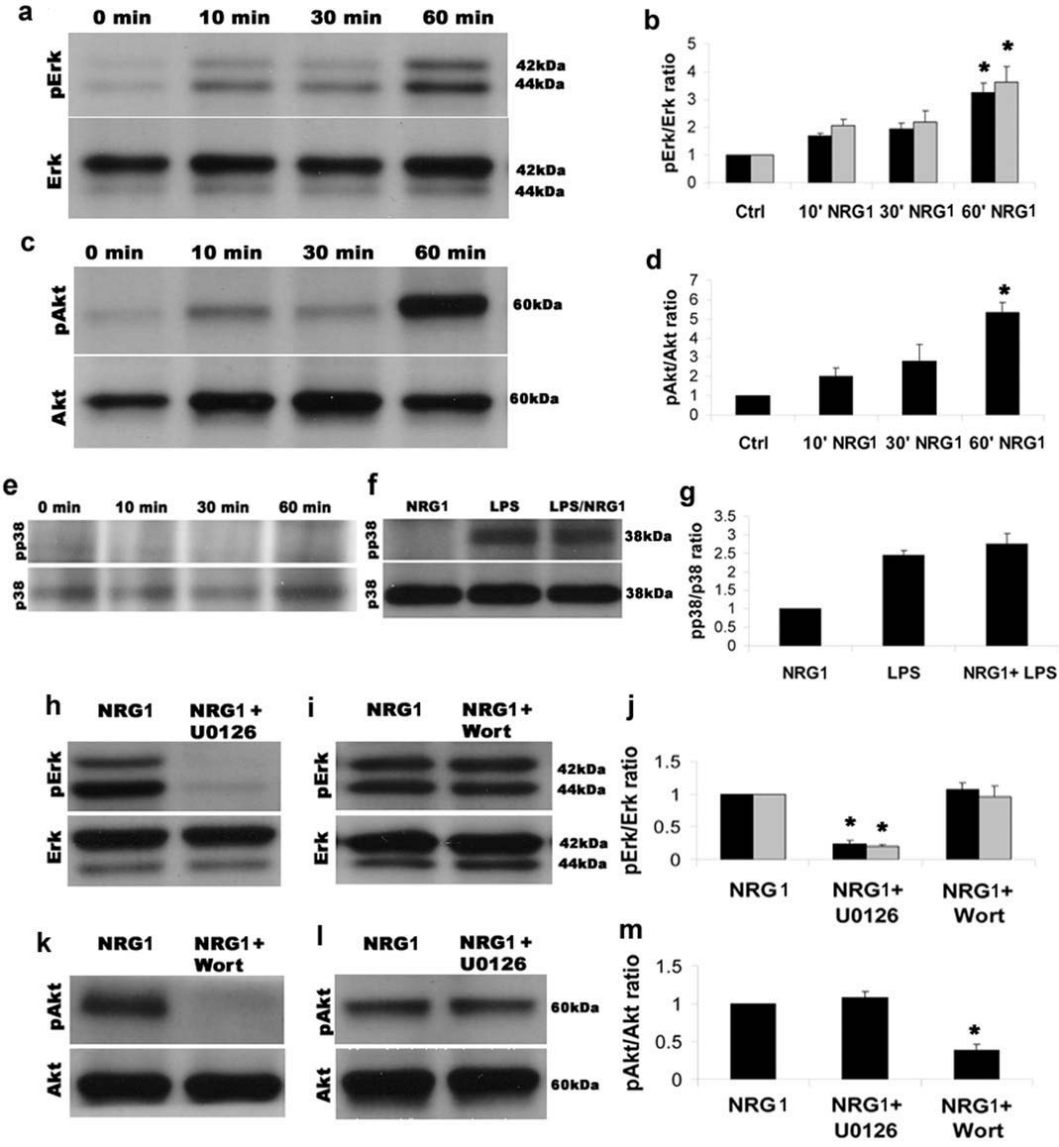
NRG1 treatment to microglial cells induced phosphorylation of ERK1/2 and Akt without activating p38MAPK. **a** and **b**: Addition of NRG1 (10 nM) to resting microglial cells induced the phosphorylation of ERK1/2 as assessed by Western Blots. A representative membrane for one experiment is shown in **a**. In **b** we show the time-course of ERK1 (black bars) and ERK2 (grey bars) phosphorylation after NRG1 treatment (ratio of phospho-ERK over total ERK). There was a significant increase in ERK1 and 2 phosphorylation after 60 min of NRG1 treatment compared with resting state or control (ERK1: control *versus* 60 min NRG1 treatment *P* = 0.02 one-way ANOVA on ranks, ERK2: control *versus* 60 min NRG1 treatment *P* = 0.003 one-way ANOVA on ranks, *n* = 4). **c** and **d**: In the same way we assessed Akt phosphorylation after NRG1 treatment. In (c) a representative membrane of one experiment is shown. In (d) we show the time-course of Akt phosphorylation (ratio of phospho-Akt over total Akt) after NRG1 treatment of three independent experiments. There was a significant increase in Akt phosphorylation after 60 min of NRG1 treatment compared with resting state or control (*P* = 0.002, one-way ANOVA, Bonferroni *post hoc* test). **e** and **f**: NRG1 treatment in microglia did not elicit phosphorylation of p38MAPK (*P* = 0.7, *t*-test, *n* = 3). In **e** we show the time-course after NRG1 treatment where no phosphorylation of p38MAPK was seen. We tested if NRG1 could enhance p38MAPK phosphorylation of LPS treated microglia but could not observe any increase in phospho-p38 when treating LPS primed microglia with NRG1. In (f) a representative membrane is shown, in (g) we show quantification of phospho-p38 over p38 of 3 independent experiments (NRG1 *vs.* LPS or LPS+NRG1 *P* = 0.003, LPS *vs.* LPS+NRG1 *P* = 0.5, one-way ANOVA, Bonferroni *post hoc* test, *n* = 3). In **h**–**j** we show that only the MEK inhibitor U0126 could block ERK1 and 2 phosphorylation (h) and phosphorylation of ERK remained the same when cells were treated with the PI3K inhibitor Wortmannin (i). Quantification of three independent experiments is shown in j (for ERK 1 and 2 the phospho/total ratio was significantly different between NRG1 treated cells compared with NRG1 plus U0126 *P* < 0.05 but not between NRG1 and NRG1 plus Wortmannin, one-way ANOVA on rank, Dunn's Method). In **k–m** we show that only Wortmannin could block Akt phosphorylation (k) and U0126 did not affect Akt activation (l). Quantification of three independent experiments is shown in m (for Akt the phospho/total ratio was significantly different between NRG1 with NRG1 plus Wortmannin *P* = 0.04, but not between NRG1 and NRG1 plus U0126, ANOVA on Ranks, Dunn's method) *P < 0.05 Wort = Wortmannin. Error bars represent ± SEM.

To elucidate which intracellular pathways are involved in NRG1 mediated effects on microglia we treated primary cultures of microglial cells with NRG1 and investigated a number of key signaling pathways within these cells. The MAPK pathway is activated by a number of different growth factors (including NRG1) and has important roles in cellular proliferation and differentiation (Di Segni et al., 2006; Nakaoka et al., 2007; Neve et al., 2002). We therefore studied two MAPK pathways: ERK and P38. As shown with Western Blots resting microglia expressed a very low level of ERK phosphorylation and no detectable p38MAPK phosphorylation in their resting state. On addition of NRG1 10 nM (a dose which in a number of different assays we have found to be optimum in regulating microglial function) to microglial cultures phosphorylation of both isoforms of ERK (1 and 2) was robustly observed (Fig. 1a,b control *vs.* NRG1 60 min ERK1: *P* = 0.02, ERK2: *P* = 0.003 one-way ANOVA on ranks, *n* = 4). By contrast, p38MAPK was not phosphorylated in response to NRG1 treatment (Fig. 1e). LPS acting via TLR4 has been shown to activate p38MAPK (Clark et al., 2006; Lehnardt et al., 2003) and we confirmed this (Fig. 1f). We also found no potentiation of p38MAPK activation by NRG1 when cells were primed with LPS (1 μg/mL) (Fig. 1e–g, LPS *vs.* LPS + NRG1 *P* = 0.5, one-way ANOVA, Bonferroni *post hoc* test, *n* = 3). The PI3K/AKT pathway has been demonstrated to be activated by NRG1 in a number of different cell types (Flores et al., 2000; Fukazawa et al., 2003; Li et al., 2001; Maurel and Salzer, 2000) and is important for cellular migration, and in some contexts for survival. This pathway is also activated in microglia as addition of NRG1 to these cells led to phosphorylation of Akt (Fig. 1c,d, control *vs.* NRG1 60 min, *P* = 0.002, one-way ANOVA, Bonferroni *post hoc* test).

The MEK/ERK1/2 and PI3K/Akt pathways (but not p38 MAPK) are therefore downstream of NRG1 signaling in microglial cells. We subsequently explored which of these pathways were involved in regulating different aspects of microglial function in response to NRG1. For this purpose we used two different kinase inhibitors (Wortmannin which is a specific covalent inhibitor of PI3K the kinase upstream of Akt, and U0126 which blocks the upstream ERK1/2 kinase, MEK1/2). We initially determined that these inhibitors had demonstrable efficacy in selectively blocking these signaling pathways in microglia. Resting microglial cells were pre-treated with the different inhibitors for one hour prior to addition of NRG1 (for 1 further hour) after which we assessed protein phosphorylation using Western Blots. Phosphorylation of ERK1 and 2 was selectively blocked by U0126 and not by Wortmannin (Fig. 1h–j, *P* < 0.05, one-way ANOVA on rank, *n* = 3). On the other hand, phosphorylation of Akt was selectively inhibited by Wortmannin and not by U0126 (Fig. 1k–m, *P* = 0.04, one-way ANOVA on rank, *n* = 3)

### The Stimulation of Microglial Proliferation by NRG1 was Dependant on the MEK/ERK1/2 Pathway

**Fig. 2:**
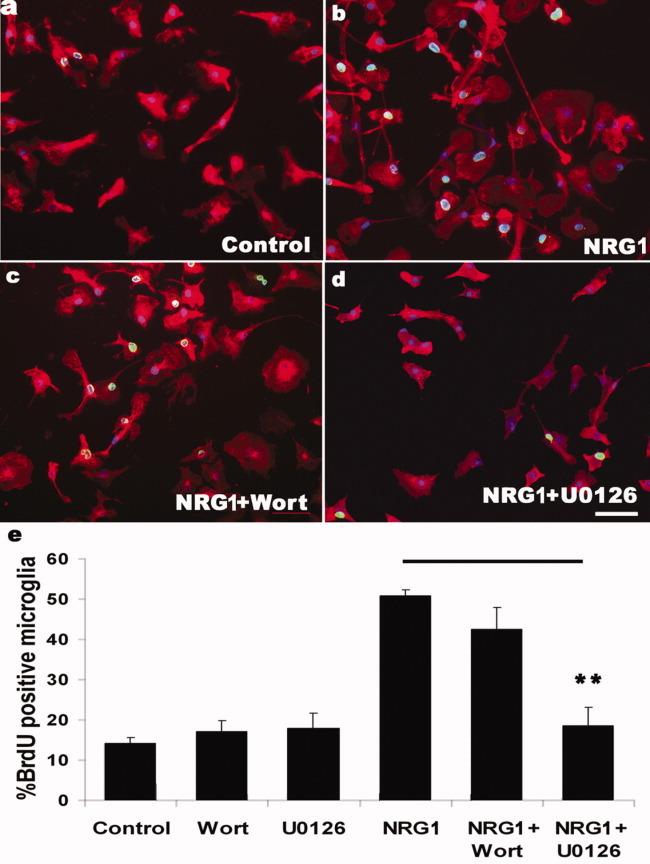
NRG1 effect on microglial proliferation was dependant on MEK/ERK1/2 pathway. Proliferation was assessed by incubating microglia in medium supplemented with 5% FBS for 3 days and pulse-labeling for 16 hours with BrdU 10 μM. Microglia, were fixed and stained (Iba1 to label microglial cells in red, DAPI to label nuclei in blue, and BrdU to label proliferating nuclei in yellow). NRG1 10 nM treatment significantly increased the proportion of BrdU-positive microglial nuclei compared with control (**a** and **b**). This effect of NRG1 was significantly inhibited when cells were treated with the MEK inhibitor U0126 (**d**) but not when cells were treated with the PI3K inhibitor Wortmannin (**c**). In (**e**) we show quantification of four independent experiments. (NRG1 treatment compared with NRG1 plus U0126 *P* < 0.001, NRG1 compared with NRG1 plus Wortmannin *P* = 0.547, one-way ANOVA, Bonferroni *post hoc* test). Note that both inhibitors by their own did not change baseline levels of proliferation. Wort = Wortmannin. Scale bar: 50 μm. Error bars represent ± SEM.

We previously showed that NRG1 is a potent proliferative factor to microglia (Calvo et al., 2010). To explore which intracellular pathway was involved in this pro-mitotic effect we used the inhibitors described above. Microglial cells were cultured in medium supplemented with 5% FBS and cell proliferation in response to NRG1, with and without the kinase inhibitors was quantified using pulse labelling with BrdU. NRG1 treatment increased the proliferation of microglia from 14% (baseline levels) to 51%. This effect was dependent on phosphorylation of ERK1/2 as it could be inhibited by the MEK inhibitor U0126 but not by the PI3K inhibitor Wortmannin (Fig. 2, NRG1 treatment alone compared with NRG1 plus U0126 *P* < 0.001, NRG1 *vs.* NRG1 plus Wortmannin *P* = 0.54, one-way ANOVA, Bonferroni *post hoc* test, *n* = 3–4).

### The Survival Effect of NRG1 in Microglia was Dependant on ERK1/2 and Akt Activation

**Fig. 3:**
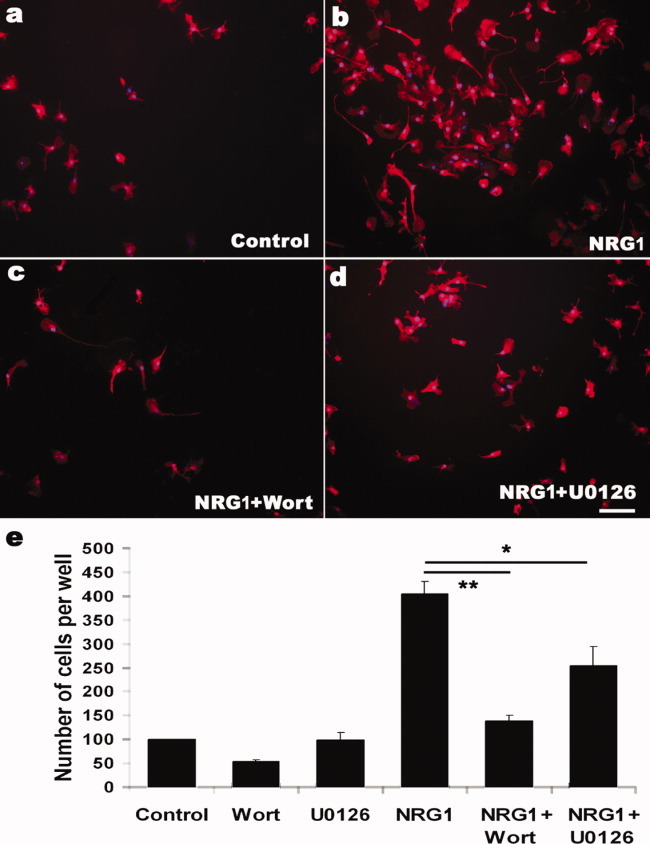
NRG1 effect on microglial survival was dependant on PI3K/Akt and MEK/ERK pathways**.** Microglial survival was assessed by incubating the cells in “starving conditions” (i.e. medium without FBS) for 3 days. Microglia, were fixed and stained (Iba1 to label microglial cells in red and DAPI to label nuclei in blue). NRG1 10 nM treatment significantly increased the number of cells per well compared with control (**a** and **b**). This effect of NRG1 was significantly inhibited when cells were treated with the PI3K inhibitor Wortmannin (**c**) and with the MEK inhibitor U0126 (**d**). In (**e**) we show quantification of five independent experiments. (NRG1 *vs.* NRG1 + Wortmannin *P* < 0.001, NRG1 *vs.* NRG1 + U0126 *P* = 0.01, one-way ANOVA, Bonferroni *post hoc* test). Note that both inhibitors by their own did not change baseline levels of survival. Wort = Wortmannin. Scale bar: 100 μm. Error bars represent ± SEM. [Color figure can be viewed in the online issue, which is available at wileyonlinelibrary.com.]

NRG1 can promote survival of microglia when these cells were left in serum free medium (a condition that normally provokes apoptotic cell death). After 72 hours of incubation in the absence of serum we assessed microglial numbers in the presence of NRG1 and the different kinase inhibitors. As previously shown NRG1 promoted microglial survival (see Fig. 3) and this effect could be blocked by adding the PI3K inhibitor Wortmannin and to a lesser extent the MEK inhibitor U0126 (NRG1 *vs.* NRG1 plus Wortmannin *P* < 0.001, NRG1 *vs.* NRG1 plus U0126 *P* = 0.01, one-way ANOVA, Bonferroni *post hoc* test, *n* = 4–5). This indicates that the PI3K/Akt and the MEK/ERK1/2 pathways are responsible for the survival-promoting effects of NRG1.

### The Chemotactic Effect of NRG1 in Microglia was Dependant on Akt Activation

**Fig. 4:**
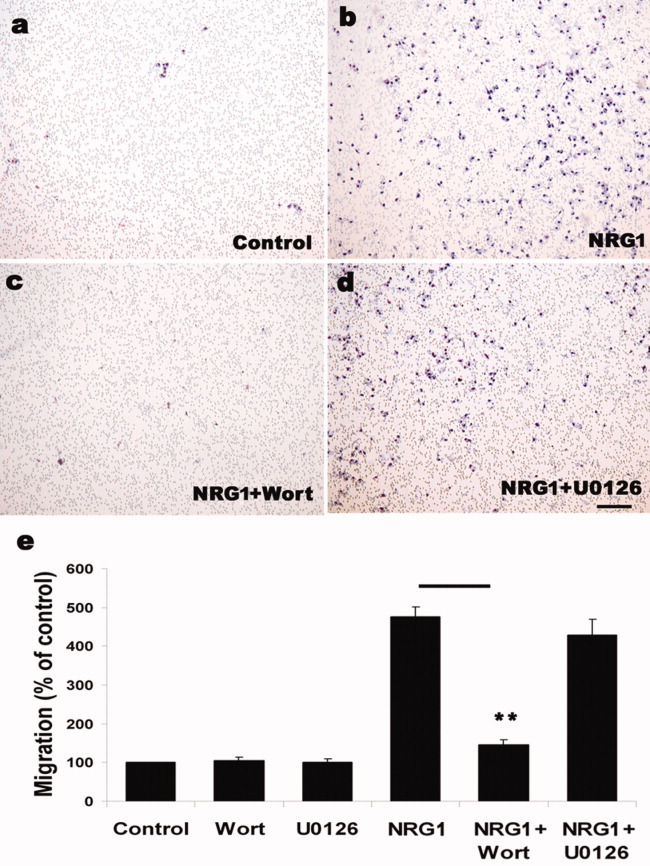
NRG1 effect on microglial chemotaxis was dependant on PI3K/Akt pathway**.** The effects of NRG1 on microglial migration were studied using a Boyden chamber. The addition of NRG1 to the lower well of the chamber (**b**) increased microglial migration to the inner membrane surface compared with control (**a**) This effect of NRG1 was significantly inhibited when cells were pretreated for 1 hour with the PI3K inhibitor Wortmannin (**c**) but not when cells were pretreated with the MEK inhibitor U0126 (**d**). In **e** we show quantification of four independent experiments. (NRG1 treatment compared with NRG1 plus Wortmannin *P* < 0.001, NRG1 treatment compared with NRG1 plus U0126 *P* = 0.8, one-way ANOVA, Bonferroni *post hoc* test). Note that both inhibitors by their own did not change baseline levels of chemotaxis. Wort = Wortmannin. Scale bar: 100 μm. Error bars represent ± SEM. [Color figure can be viewed in the online issue, which is available at wileyonlinelibrary.com.]

Microglial cells can migrate through the CNS towards a site of injury and indeed NRG1 can act as a chemotactic factor for microglia. Using a Boyden chamber in which microglia migrate through pores in a polycarbonate filter across a concentration gradient we tested which intracellular signaling pathways are involved in the chemo-attractant effect of NRG1. Pre-incubating microglia for one hour with Wortmannin before adding the cells to the Boyden chamber with a NRG1 gradient could completely block the chemoattractive effect of NRG1 (Fig. 4, NRG1 *vs.* NRG1 plus Wortmannin *P* <0.001, one-way ANOVA, Bonferroni *post hoc*). Preincubation with U0126 had no effect (*P* = 0.8) suggesting that PI3K/Akt and not MEK/ERK1/2 pathway is involved in generating the chemotactic response to NRG1.

### Intrathecal NRG1-Induced Sustained ERK1/2 Phosphorylation but Transient Akt Activation in Spinal Microglia of Naive Animals

**Fig. 5:**
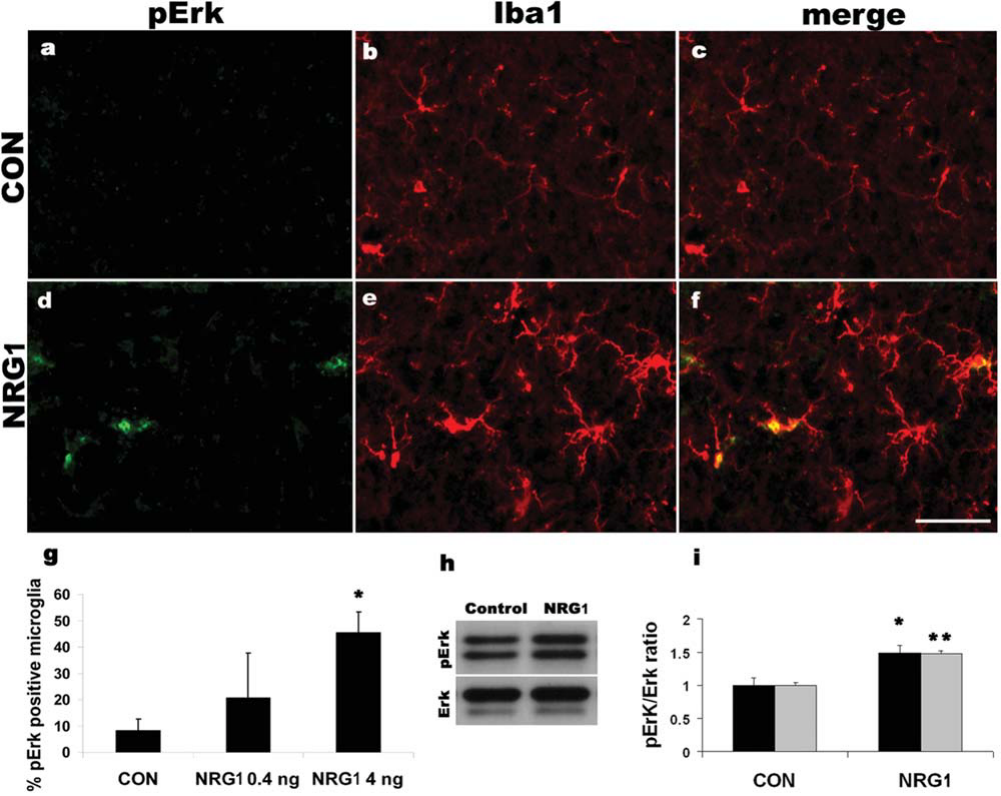
Intrathecal administration of NRG1 for 4 consecutive days induced ERK1/2 phosphorylation in spinal microglia of naive animals. Representative sections are shown of control (saline in **a–c**) and NRG1 (in **d**–**f**) immunostained with an antibody that recognises the phosphorylated form of ERK1/2 (green) and Iba1 (red). In (**g**) we show quantification of the percentage of microglia (Iba1 positive cells) that were phospho-ERK positive. A significant increase in microglia presenting phospho-ERK was found when comparing control *vs.* NRG1 4 ng (*P* < 0.05, one-way ANOVA on ranks). We confirmed these results using WB. Phosphorylation of ERK 1 and 2 was significantly increased after NRG1 intrathecal injections (**h**). In (**i**) we showed the quantification of the ratio of phosphorylated to total ERK 1 and 2 (control *vs.* NRG1 ERK1 *P* = 0.02, ERK2 *P* < 0.001, *t*-test, *n* = 4 per group) **P < 0.001; *P < 0.05. Scale bar: 50 μm. Error bars represent ± SEM.

To investigate the intracellular signaling involved in mediating NRG1 effects on microglia *in vivo* we administered this molecule intrathecally (0.4 or 4 ng administered daily for 4 days) to naive animals and used immunohistochemistry and western blot analysis to identify the intracellular pathways activated. We observed no associated morbidity with the repeated i.t. injections (there was no change in general well being or body weight of the animals in all groups; animals from the saline group presented unchanged mechanical and cold sensitivity throughout the study). At 4 days after repeated NRG1 injections this molecule induced an increase in microglial numbers in the dorsal horn and these cells expressed the phosphorylated form of ERK1/2. Very few microglia in the dorsal horn of control animals presented phospho-ERK immunoreactivity (approximately 10%) compared with NRG1 (4 ng) treated animals in which 45% of dorsal horn microglial were phospho-ERK positive (Fig. 5a–g, control *vs.* NRG1 4 ng *P* < 0.05, one-way ANOVA on ranks). We confirmed these results by performing Western blots analysis which showed a significant increase in ERK1/2 phosphorylation within the spinal dorsal horn of animals treated with NRG1 (4 ng) compared with control (Fig. 5h,i, control *vs.* NRG1 ERK1 *P* = 0.02, ERK2 *P* < 0.001, *t*-test, *n* = 4 per group).

We also studied the p38MAPK and PI3K/Akt pathways. Following 4 days of repeated intrathecal treatment with 4 ng NRG1 (a dose which leads to increased microglial numbers and pain related behaviour see below) we found very scarce phospho-p38 immunoreactivity in microglia which was not significantly different from control (*P* = 0.6, Mann-Whitney Rank Sum Test, *n* = 4 per group, Supp. Info. Fig. 1a–g). The Western blot analysis of the spinal cord of these animals showed no change in p38MAPK phosphorylation after NRG1 treatment (*P* = 0.6, *t*-test, *n* = 4 per group, Supp. Info. Fig. 1h,i).

Similarly, at four days (96 hours) after repeated NRG1 injections very few microglia presented phospho-Akt immunoreactivity which was not significantly different from control (Supp. Info. Fig. 2). This was confirmed by Western Blots in which we found no difference in Akt phosphorylation (*P* = 0.5, *t*-test, *n* = 4 per group, Supp. Info. Fig. 2h,i). As we had previously found that the PI3K/Akt pathway is activated by NRG1 in microglial cells *in vitro* we further investigated the involvement of this pathway at an earlier time points after NRG1 injection *in vivo*. We found a transient and small activation of this pathway which peaked at 6 hours after NRG1 injections and then returned to control (there was a significant increase in the percentage of microglia that were phospho-Akt positive; from 0.24% in control to 2.71% in NRG1 6 hours, *P* < 0.001, one-way ANOVA, Bonferroni *post hoc* test, *n* = 4, Supp. Info. Fig. 2a–g). Therefore the key signaling pathway activated within microglia following NRG1 treatment *in vivo* is the MEK/ERK pathway and we subsequently focused on this.

### Inhibition of MEK1/2 Could Reverse NRG1-Induced Microgliosis and Mechanical and Cold Pain-Related Hypersensitivity

**Fig. 6:**
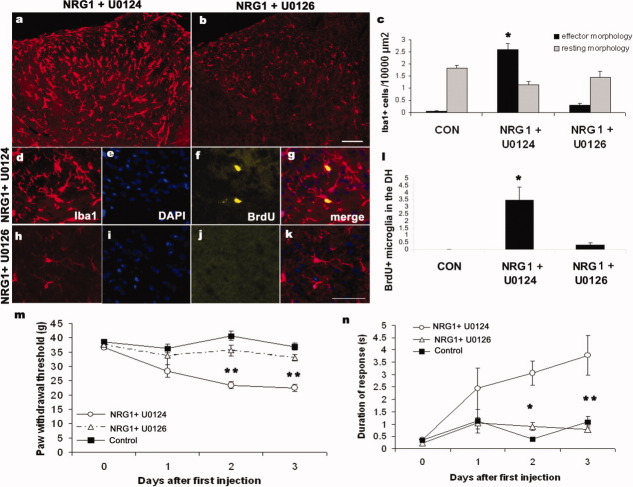
Inhibition of MEK1/2 could reverse NRG1-induced microgliosis and mechanical and cold pain-related hypersensitivity. NRG1 was administered intrathecally (4 ng given daily for 3 days) together with the MEK inhibitor U0126 (10 μg) or the inactive analogue U0124 (10μg). **a** and **b**: Dorsal horn of animals treated with NRG1 + U0124 (in a) or NRG1 + U0126 (in b) immunostained with Iba1. Note that U0126 but not U0124 could prevent NRG1 associated increase in numbers of microglia with an effector morphology. In **c** quantification of this response is shown. In **d**–**k** we assessed proliferation (pulse labeling with BrdU) after injections (Iba1 is shown in red, DAPI in blue, BrdU in yellow and in the last panel merged images are shown). d–g: Dorsal horn microglia from a NRG1 + U0124 treated animal. h–k: Dorsal horn microglia from a NRG1 + U0126 treated animal. In (**l**) quantification of all BrdU-positive microglia in the dorsal horn is shown. Again, U0126 but not U0124 prevented NRG1 induced increase in microglial proliferation. Mechanical (shown in **m**) and cold (shown in **n**) pain related hypersensitivity developed after NRG1 injections which were reversed by U0126 but not by U0124. Scale bars: a and b: 100 μm, d–k: 50 μm. *P <0.05, **P < 0.001. Error bars represent ± SEM.

We next tested the hypothesis that MEK/ERK1/2 pathway was necessary for NRG1 signaling *in vivo*. For this purpose we intrathecally administered the MEK inhibitor U0126 (10 μg) or its inactive analogue U0124 (10 μg) at the same time as NRG1 (4 ng). We repeated the i.t injection every 24 hours for 4 days. Animals underwent behavioural testing every day for 3 days and were perfused at day 4. NRG1 injections elicited microgliosis in the lumbar spinal cord. Animals injected with NRG1 plus the inactive analogue U0124 showed an increase in microglial numbers, increased proliferation of these cells (as shown with BrdU labeling) and in addition the morphology of these cells changed with cellular hypertrophy and process retraction. We found that the NRG1 induced microgliosis was dependant on the MEK/ERK1/2 pathway as inhibiting ERK1/2 phosphorylation by MEK (using U0126) could prevent microgliosis (Fig. 6a–l). When NRG1 was injected with the inactive analogue U0124 there was a significant increase in the number of microglia with an effector morphology (*P* < 0.001) and which were BrdU labeled (*P* = 0.001) compared with saline injections. These effects were virtually completely blocked when NRG1 was injected together with the MEK inhibitor U0126 which resulted in no significant difference from the saline group. We used one-way Analysis of Variance on Ranks in both comparisons. Intrathecal NRG1 is pronociceptive and in these animals it elicited mechanical and cold pain related hypersensitivity. The MEK inhibitor U0126 could completely prevent NRG1 induced mechanical hypersensitivity (Fig. 6m,n. Mechanical hypersensitivity was significantly different between NRG1 + U0124 *vs.* NRG1 + U0126 at day 2 and day 3 (both *P* < 0.001). Cold hypersensitivity was also significantly different between these groups at day 2 (*P* = 0.004) and day 3 (*P* < 0.001) (for both comparisons we used RM two-way ANOVA Bonferroni *post hoc*, *n* = 8 per group).

### Blocking NRG1/erbB2 Signaling After L5 Spinal Nerve Ligation Reduced ERK1/2 Phosphorylation in Spinal Cord Microglia

**Fig. 7:**
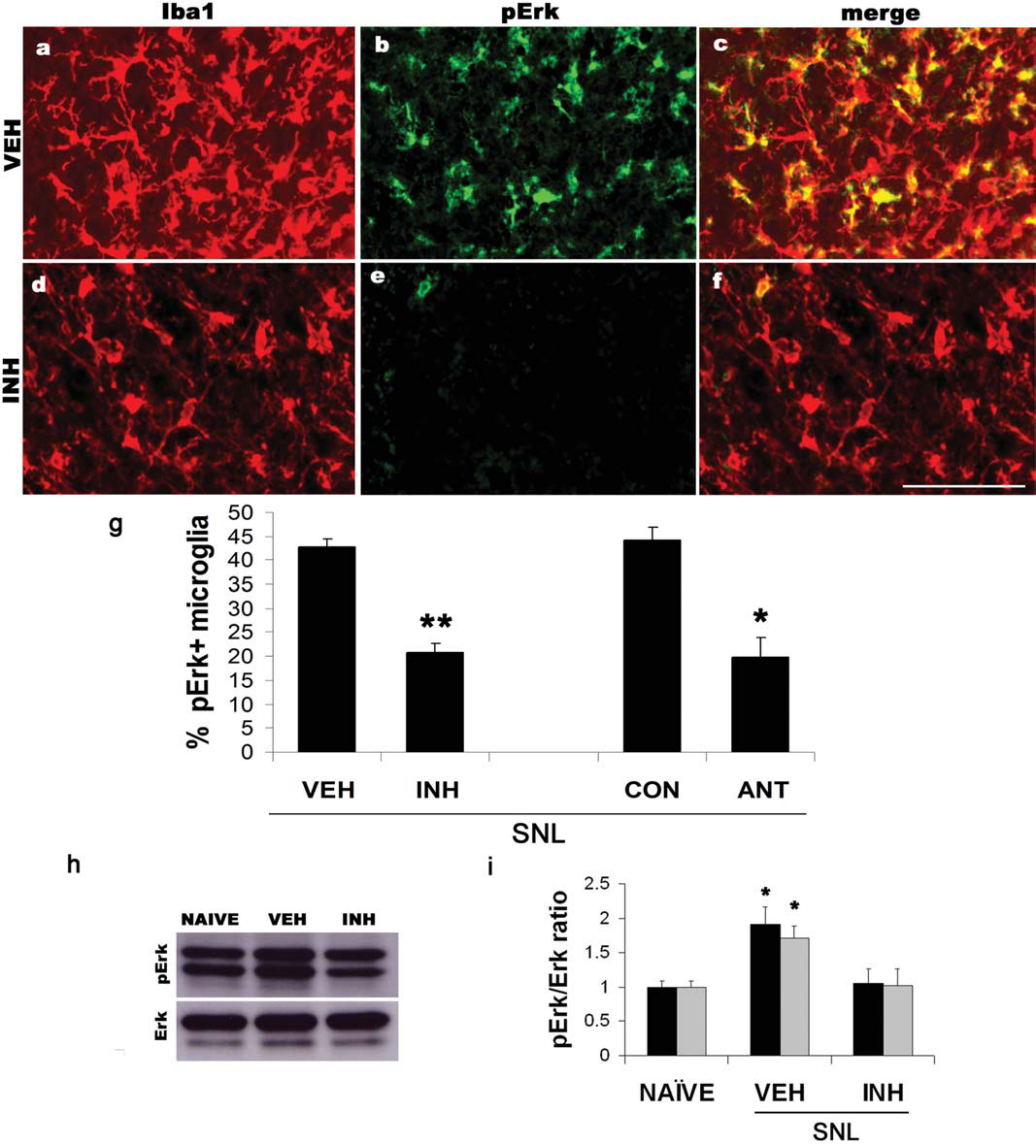
Blocking NRG1-erbB signaling after L5 SNL reduced ERK1/2 phosphorylation in spinal cord microglia. Using immunohistochemistry (Iba1 in red, phospho-ERK in green) we observed that ERK was phosphorylated in spinal microglia following SNL. In **a**–**c**, a representative section of the ipsilateral dorsal horn of a vehicle treated animal is shown. In **d**–**f**, a representative section of an animal treated with PD168393 (erbB receptor inhibitor) is shown. Note the reduction of phospho-ERK positive microglia when this inhibitor is administered. We also used a NRG1 sequestering molecule (HBD-S-H4) that was injected intrathecally following L5 SNL and found similar results. In (**g**) we show the quantification of the percentages of phospho-ERK positive microglia in both experiments (vehicle *vs.* erbB inhibitor *P* < 0.001, *t*-test, *n* = 4; control *vs.* NRG1 antagonist *P* = 0.006, *t*-test, *n* = 4). To confirm these results we used WB analysis of the ipsilateral dorsal horn of animals treated with the erbB inhibitor. A representative blot is shown in (**h**) in where an increase in phosphorylated ERK1/2 is seen after nerve injury which was greatly decreased when using the erbB receptor inhibitor. In (**i**) we show quantification of the WB of four animals per group (vehicle *vs.* inhibitor: ERK1 and 2 *P* = 0.04, one-way ANOVA on ranks, Bonferroni *post hoc*) *P < 0.05, **P < 0.001. Scale bar: 50 μm. Error bars represent ± SEM. VEH: vehicle, INH: erbB2 inhibitor (PD168393), ANT: HBD-S-H4.

We next studied whether the ERK pathway is downstream of NRG1-erbB signaling in a nerve injury model which results in a robust microgliosis [L5 spinal nerve ligation (Kim and Chung, 1992)]. As previously shown by others (Zhuang et al., 2005) we found that the proportion of microglia which expressed phospho-ERK increased from a naive level of around 8% to 45% 3 days after such injury. To test if the erbB receptor kinases activated by NRG1 were upstream of ERK phosphorylation we administered the specific erbB receptor blocker PD168393 [which blocks the ATP binding site of the receptor (Bose et al., 2006) and we have previously found effective in inhibiting NRG1 signaling *in vivo* (Calvo et al., 2010)] via a continuous intrathecal infusion following spinal nerve ligation. At day 3 after spinal nerve ligation, when the microglial response is well established, we found that the erbB receptor inhibitor PD168393 dramatically reduced the percentage of phospho-ERK immunoreactive microglia in the ipsilateral dorsal horn from approximately 45% in vehicle to 20% in erbB inhibitor infused animals (Fig. 7a–g, *P* < 0.001 *t*-test, *n* = 4 per group). PD168393 will not only block NRG1 signaling but through inhibition of erbB1 may also interfere with EGF signaling. For this reason an alternative strategy was also employed to block NRG1-erbB signaling. We used a molecule (HBD-S-H4) to sequester endogenous NRG1. This is a fusion protein consisting of the soluble ectodomain of erbB4, which has high affinity for NRG1, and a heparin-binding domain, which helps target the molecule to the same heparan-sulfate rich cell surfaces that bind NRG1. This molecule has previously been shown to be highly effective *in vitro* and *in vivo* (Calvo et al., 2010; Ma et al., 2009). Intrathecal administration of HBD-S-H4 (3 μg, i.t. injection) significantly reduced the percentage of phospho-ERK immunopositive microglia (form approximately 44% in control to 19% in NRG1 antagonist treated animals, *P* = 0.006, *t*-test, *n* = 4, Fig. 7g).

To confirm these results we performed Western blots analysis to detect the ratio of phosphorylated to total ERK 1 and 2 in the dorsal horn of PD168393 *versus* vehicle treated animals 3 days after spinal nerve ligation. The phosphorylation of ERK 1 and 2 presented a twofold increase following nerve injury but this change was reversed by treatment with PD168393 (Fig. 7h,i, vehicle *vs.* inhibitor: ERK1 and 2 *P* = 0.04, one-way ANOVA on ranks, Bonferroni *post hoc*, *n* = 4 per group).

Interestingly we have previously found that disrupting the NRG1-erbB signaling pathway after SNL could reduce the total number of microglial cells expressing the phosphorylated form of p38MAPK (Calvo et al., 2010). This seems at odds with the fact that we could not find activation of p38 MAPK by NRG1 either *in vitro* or *in vivo*. When we further analyzed this data however we have found that the percentage of microglia expressing phospho-p38MAPK did not change with erbB inhibition. In sham animals 29% of microglia were phospho-p38MAPK immunoreactive, this percentage increased after peripheral nerve injury to 83% (vehicle animals) and remained the same with erbB inhibition (74%, *P* = 0.27, one-way ANOVA, Bonferroni *post hoc* test, *n* = 3–5). The reduction in total pp38 immunoreactive microglia following erbB inhibition is likely to reflect a reduction in the number of microglia within the dorsal horn rather than a direct modulation of this pathway by NRG1.

### Microglial Proliferation After L5 Spinal Nerve Ligation was Decreased by Blocking MEK1/2 Pathways

**Fig. 8:**
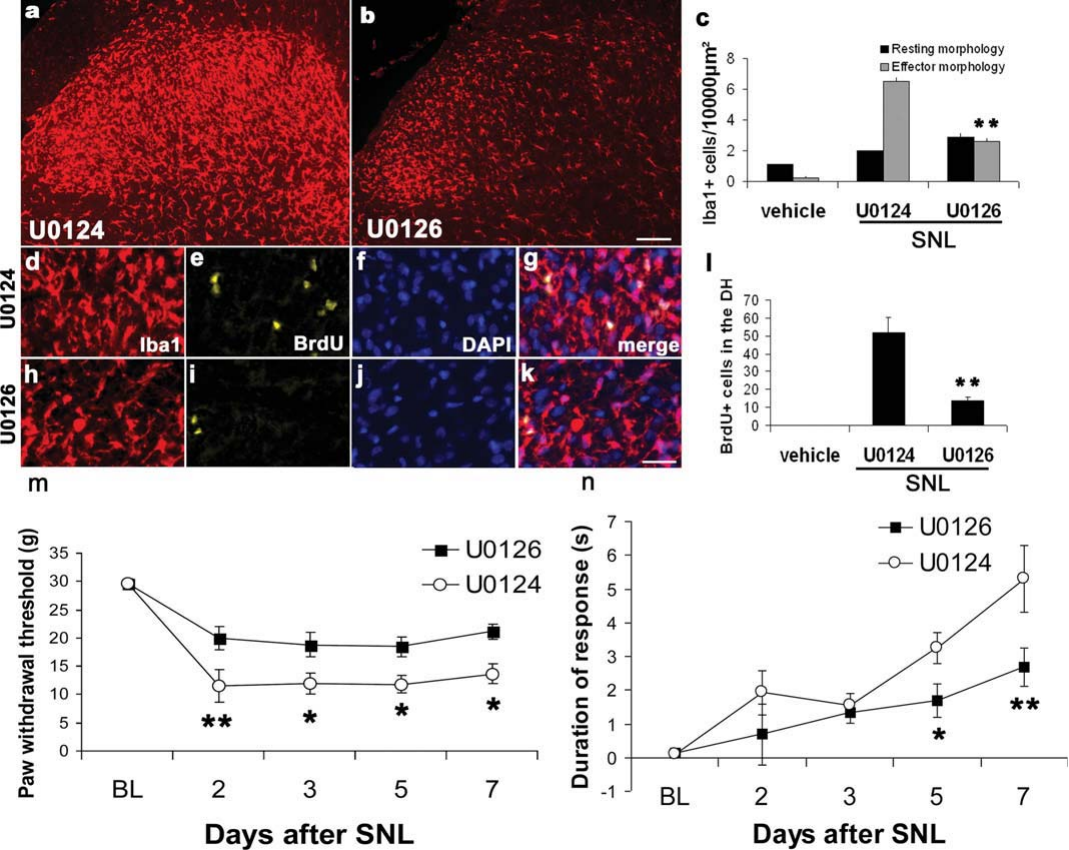
Microgliosis and mechanical and cold hypersensitivity following L5 SNL were significantly reduced by blocking MEK1/2 pathways. Animals underwent L5 SNL and were injected intrathecally daily with a 10 μg dose of the MEK inhibitor U0126 or its inactive analogue U0124. Three days after SNL animals treated with the inactive analogue U0124 demonstrated a dramatic increase in the number of dorsal horn microglia showing an effector morphology compared with naïve animals injected with saline. This microgliosis was significantly reduced when animals received the MEK inhibitor U0126. In (**a**) (SNL + U0124) and (**b**) (SNL + U0126) we show representative sections of the L5 spinal cord of these animals stained with Iba1 to label microglia. In (**c**) we show the quantification (*n* = 4 per group, *P* < 0.001 SNL + U0124 *vs.* SNL + U0126, one-way ANOVA, Bonferroni *post hoc* test). Similarly, increased microglial proliferation was found in SNL animals that were treated with the inactive MEK inhibitor and it was greatly reduced (by more than 70%) in animals treated with U0126. In **d**–**k**, we show representative section of L5 spinal cord immunostained for Iba1 (red), BrdU (yellow), and DAPI (blue). Panels d–g show an inactive analogue (U0124) treated animal and panels h–k show a MEK inhibitor treated animal, both 3 days after L5 SNL. Quantification of BrdU-positive nuclei in the dorsal horn is shown in (**l**) (*n* = 4 per group, *P* < 0.001 SNL + U0124 *vs.* SNL + U0126, one-way ANOVA, Bonferroni *post hoc* test). Mechanical (shown in **m**) and cold (shown in **n**) pain related hypersensitivity developed after L5 SNL which were partially prevented by U0126 but not by U0124 (**P < 0.001, *P < 0.05, RM two-way ANOVA, Bonferroni *post hoc* test, *n* = 7–8 for m and n). Scale bars, a and b 100 μm, c–j 25 μm.

We have previously shown that NRG1-erbB signaling induces microglial proliferation after spinal nerve ligation. To test wherever this *in vivo* effect was dependant on MEK/ERK1/2 pathways we administered the MEK inhibitor U0126 or its inactive analogue U0124 intrathecally via repeated lumbar punctures following nerve injury. We pulse labeled the animals with BrdU to assess cell proliferation. Microgliosis was dramatically reduced by the MEK inhibitor (Fig. 8, *P* < 0.001 comparing SNL + U0124 *vs.* SNL + U0126, one-way ANOVA, Bonferroni *post hoc*, *n* = 4 per group). The time point of 3 days postinjury was used because microglial proliferation is maximal at this time (Echeverry et al., 2008) and thus we found a marked increase in the rate of microglial proliferation within the dorsal horn of the spinal cord. Interestingly we observed that blocking MEK with U0126 could reduce microglial proliferation horn by 73% (Fig. 8, *P* < 0.001 comparing SNL + U0124 *vs.* SNL + U0126, one-way ANOVA, Bonferroni *post hoc*, *n* = 4 per group)

### Inhibition of MEK1/2 Reduced the Mechanical and Cold Pain-Related Hypersensitivity Following Peripheral Nerve Injury

Inhibition of the MEK/ERK pathway has been shown previously to reduce mechanical hypersensitivity after spinal nerve ligation (Obata et al., 2004; Zhuang et al., 2005). We tested if inhibiting this pathway, which could reduce microglial proliferation, would also reduce mechanical and cold pain related hypersensitivity after nerve injury. Rats underwent L5 SNL and received daily intrathecal injections with the MEK inhibitor U0126 or the inactive analogue U0124. Animals receiving U0124 developed mechanical and cold hypersensitivity. The MEK inhibitor could partially prevent mechanical hypersensitivity (Fig. 8m, *P* < 0.001 at day 2, *P* = 0.001 at day 3, *P* = 0.01 at day 5, and *P* = 0.002 at day 7). Cold hypersensitivity was also significantly different between these groups at day 5 (*P* = 0.03) and day 7 (*P* < 0.001) (for both comparisons we used RM two-way ANOVA Bonferroni *post hoc*, *n* = 7–8 per group).

## DISCUSSION

NRG1 has recently been identified as a pro-nociceptive growth factor which is expressed by primary afferents, released within the dorsal horn following nerve injury and which promotes microgliosis and hence the development of neuropathic pain. Here we show that NRG1 activates both the MEK/ERK and PI3K/AKT pathways in microglia *in vitro*. The former modulates proliferation and the latter chemotaxis of cultured microglia, both appear to have a role in survival. Intrathecal treatment with NRG1 *in vivo* produced activation of the MEK/ERK pathway and this was critical for the development of microglial proliferation and pain related behaviour. Following nerve injury ERK was activated within microglia downstream of NRG1-erbB signaling and this drives microglial proliferation and the development of neuropathic pain.

### Microglia and Neuropathic Pain: The Role of NRG1

Peripheral nerve injury results in a dramatic increase in the number of microglia within the spinal cord through proliferation and migration of resident cells (Ajami etal., 2007; Echeverry et al., 2008; Raivich et al., 1994). Microglia have receptors for and respond to injury signals such as cytokines and chemokines (e.g. CCL2, Fractalkine, and IFN-γ), complement components and purines (Clark et al., 2007; Griffin etal., 2007; Tanga et al., 2005; Tsuda et al., 2003, 2009; Thacker et al., 2009; Verge et al., 2004; Zhang et al., 2007). Microglia switch into a proinflammatory phenotype secreting cytokines, nitric oxide and BDNF which enhance the excitability of dorsal horn neurons (Coull et al., 2005; Kawasaki et al., 2008). Microgliosis at the level of the brainstem can contribute to enhanced descending pain facilitation following nerve injury (Wei et al., 2008). This excessive pro-inflammatory microglial response to which NRG1 contributes is clearly deleterious as it promotes the development and maintenance of neuropathic pain (Calvo et al., 2010; Clark et al., 2007; Coull et al., 2005; Ledeboer et al., 2005; Tsuda et al., 2003; reviewed in Ren and Dubner, 2008).

ErbB receptor activation by NRG1 results in the stimulation of a number of intracellular signaling pathways including the PI3K/AKT, MEK/ERK, and p38MAPK pathways (Di Segni et al., 2006;Liu et al., 1999; Nakaoka et al., 2007) resulting in the induction of a variety of cellular responses including proliferation, migration, differentiation, survival or apoptosis (reviewed in Britsch, 2007). Our initial approach in investigating intracellular signaling pathways activated by NRG1 in microglia was to study the actions of this molecule on primary cultures of these cells and subsequently investigate their role in mediating the effects of NRG1 *in vivo*.

### The PI3K-AKT Pathway Mediates the Microglial Survival and Chemotactic Effects of NRG1 *In Vitro*

Akt is a downstream effector of phosphoinositide 3-kinases(PI3Ks); it has been shown to be activated in response to NRG1-erbB signaling and to increase survival in a variety of different cell types (Flores et al., 2000; Fukazawa et al., 2003; Li et al., 2001; Maurel and Salzer, 2000). Within microglia and macrophages the PI3K/AKT pathway promotes survival and regulates cell polarity, which is necessary for cell motility and directional sensing in the process of chemotaxis for instance to ATP (Adapala et al., 2010; Hirsch et al., 2000; Horvath and DeLeo, 2009; Irino et al., 2008; Ohsawa etal., 2007; Ruan et al., 2010; Weiss-Haljiti et al., 2004).

NRG1 treatment of cultured microglial cells significantly increased phosphorylation of Akt. Selective inhibition of PI3K virtually abolished the survival and the chemotactic effects of NRG1 on cultured microglia. In contrast to the findings in cultured microglia we only found transient activation of PI3K/AKT in a very small population of microglia following intrathecal treatment with NRG1 *in vivo* in a manner that did not correlate with pain related behaviour. Importantly this was at a dose of NRG1 which produces both microgliosis, and mechanical and cold pain related hypersensitivity. It is theoretically possible that a higher dose of NRG1 may have activated PI3K/Akt however the physiological relevance would have been unclear. One potential reason for these differences is the different cellular context of microglia in primary cultures *versus* whole spinal cord. However in accordance with previous reports we did observe an increase in phospho-Akt immunoreactivity within the dorsal horn at 3 days after peripheral nerve injury (Xu et al., 2007) but most of the cells expressing it were not microglia (unpublished observations). Inhibition of this pathway has been shown to reduce pain related hypersensitivity (Xu et al., 2007) and therefore PI3K/Akt pathway seems to play a role in regulating neuronal plasticity *in vivo* but it is not a key mediator of NRG1 action on microglia.

### Activation of the MEK/ERK Pathway by NRG1 Promotes Microglial Proliferation and the Development of Neuropathic Pain

Mitogen-activated kinases (MAPKs) are a family of well conserved molecules critical in intracellular signal transduction with important roles in regulating neural plasticity and the inflammatory response. They include three major members: extracellular signal-regulated kinase (ERK1/2 or p42/44 MAPK), p38, and c-Jun N-terminal kinase (JNK). Following peripheral nerve injury p38MAPK and ERK have been shown to be activated in spinal microglia and JNK is activated in astrocytes (reviewed in Ji et al., 2009).

P38 MAPK, is regarded as a stress-induced kinase as it plays a critical role in inflammatory responses (reviewed in Ji and Suter, 2007). It is important for the synthesis of several pro-inflammatory mediators by microglia such as cyclooxygenase-2, IL-1β, BDNF; and iNOS (Clark et al., 2006; Coull et al., 2005; Sung et al., 2005; Svensson et al., 2003; Trang et al., 2009). Following nerve injury P38 MAPK activation contributes to neuropathic pain development/maintenance (Jin et al., 2003; Tsuda et al., 2004). We did not however find evidence of activation of p38 MAPK by NRG1 either in cultured microglia or following intrathecal treatment *in vivo*. We have previously shown that inhibition of NRG1-erbB signaling following SNL reduces the number of microglia within the dorsal horn of the spinal cord and the total number of phospho-p38 immunoreactive microglia. However the proportion of microglia expressing phospho-p38 MAPK did not change with inhibition of the NRG1-erbB signaling. The decrease in the total number of phospho-p38 MAPK positive microglia following NRG1-erbB signaling disruption is therefore not a direct effect of NRG1 but may be due to the fact that NRG1 is required for the early proliferative response of these cells (see below).

ERK exists in two isoforms (1 and 2) which are activated by upstream kinase MEK1/2 (MKK1/2) and has been implicated in regulating the proliferation, differentiation, and survival of cells during development (reviewed in Widmann etal., 1999). ERK activation has been shown to be essential for the proliferative actions of a number of growth factors such as GM-CSF on microglia (Suh et al., 2005). ERK also mediates the release of a number of pro-inflammatory mediators such as TNF-α and NO from cultured microglial cells (Bhat et al., 1998; Hide et al., 2000; Romero-Sandoval et al., 2009; Tichauer et al., 2007). In spinal cord injury models MEK/ERK inhibition dramatically reduced microglial accumulation, IL-1β expression and the spinal release of prostaglandin E2 (Lu et al., 2007; Zhao et al., 2006, 2007). Following peripheral nerve injury ERK is activated acutely within hours in dorsal horn neurons, from day 2 postinjury in microglia and then more chronically from day 10 onwards in astrocytes (Zhuang et al., 2005). ERK signaling is also activated within microglia in the Streptozotocin induced model of diabetic neuropathy (Tsuda et al., 2008). Inhibition of the MEK/ERK pathway can reduce mechanical pain related hypersensitivity in these different models of nerve injury. Following peripheral axotomy the Src-family kinases (SFKs) are activated in spinal microglia upstream of ERK and contribute to the development of mechanical hypersensitivity (Katsura et al., 2006). This is interesting as it has been shown that the erbB signaling requires the engagement of Src kinases to trigger MAPK activation (Olayioye et al., 2001).

NRG1 produced phosphorylation of ERK1 and 2 in cultured microglial cells. Inhibition of this pathway prevented NRG1 induced proliferation *in vitro*. Intrathecal treatment with NRG1 in naïve animals induced ERK1/2 phosphorylation within spinal cord microglia. This was associated with microglial proliferation, adoption of an effector morphology, and development of cold and mechanical pain related hypersensitivity. These changes could be virtually completely reversed by MEK inhibition. Spinal nerve ligation provides a well-characterized model of nerve injury associated with a robust microgliosis (Scholz and Woolf, 2007; Tsuda et al., 2003) and development of neuropathic pain (Kim and Chung, 1992). SNL results in increased NRG1 release within the spinal cord dorsal horn (Calvo et al., 2010) and CSF (Calvo et al., unpublished observations) resulting in activation of the erbB2 receptor specifically within microglia (Calvo et al., 2010). We have found that activation of NRG1-erbB signaling in these cells following axotomy is accompanied by extensive ERK 1/2 phosphorylation. This activation of ERK signaling is downstream of the erbB receptors as receptor inhibition or sequestration of endogenous NRG1 resulted in a significant reduction in ERK1/2 phosphorylation. Inhibition of the MEK/ERK1/2 pathway following SNL reduced both microglial proliferation as well as mechanical and cold pain related hypersensitivity. ERK inhibition has also been shown to reduce established neuropathic pain related behavior within 3 hours of intrathecal injection (Zhuang et al., 2005). This rapid response would indicate that the effects of ERK inhibition are not solely related to a reduction in microglial proliferation but will also include actions such as reduced cytokine release (Zhao et al., 2007). In summary although both ERK and p38 have been shown to be activated in microglia following nerve injury only the former is directly stimulated by NRG1 signaling. There is some evidence that these signaling pathways may be activated differentially among the microglial population (Katsura et al., 2006).

## CONCLUSIONS

We conclude that peripheral nerve injury results in the release of NRG1 within the dorsal horn of the spinal cord and activation of erbB receptors on microglia. This stimulates the MEK/ERK1/2 pathway which plays a pivotal role in the microglial mitotic response and contributes to the development of neuropathic pain. This pathway offers a potential target to modulate microgliosis and may also be relevant to other forms of injury to the nervous system.
