# Supplementary figures and images for: Following Nerve Injury Neuregulin-1 Drives Microglial Proliferation and Neuropathic Pain via the MEK/ERK Pathway
Source: Glia. 2011 Jan 6;59(4):554–68. doi: 10.1002/glia.21124 (PMC3222694; doi:10.1002/glia.21124)

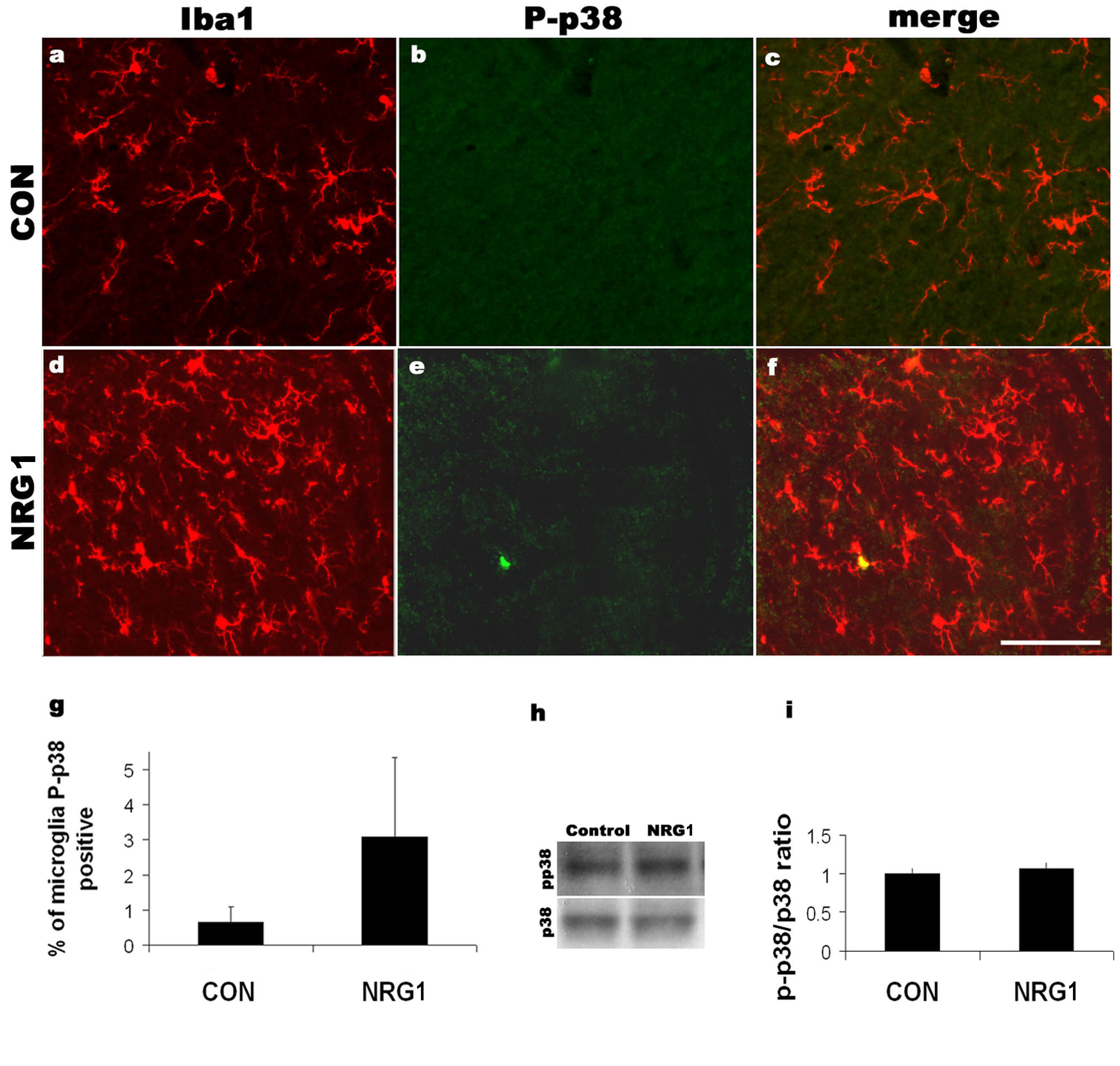

Supplement: Supplementary file 1 [file glia0059-0554-SD1.tif]

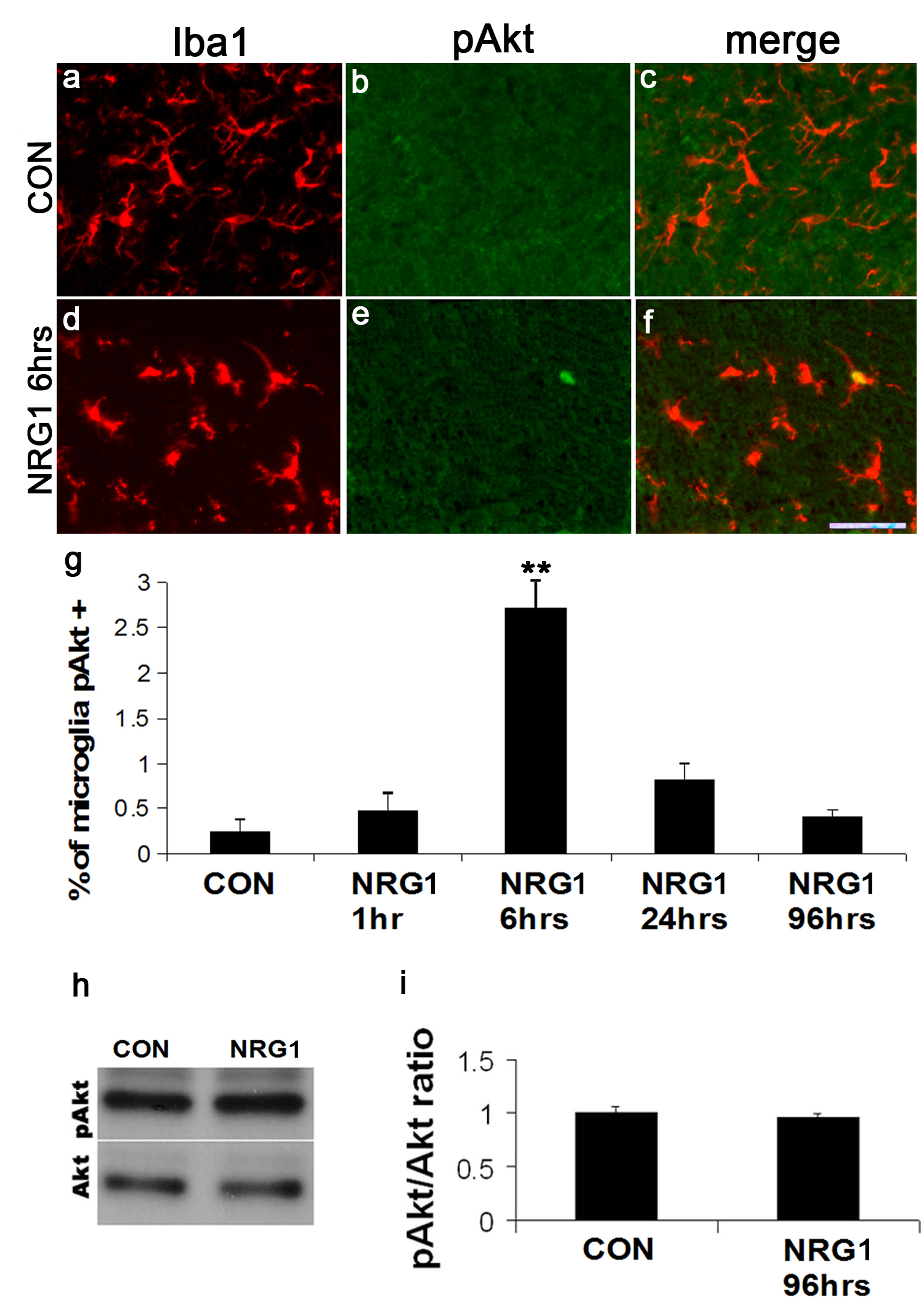

Supplement: Supplementary file 2 [file glia0059-0554-SD2.tif]
